# TEK, local perceptions of risk, and diversity of management practices of *Agave inaequidens* in Michoacán, Mexico

**DOI:** 10.1186/s13002-015-0043-1

**Published:** 2015-08-05

**Authors:** Ignacio Torres, José Blancas, Alejandro León, Alejandro Casas

**Affiliations:** Instituto de Investigaciones en Ecosistemas y Sustentabilidad (IIES), Universidad Nacional Autónoma de México (UNAM), Morelia, Michoacán Mexico; Centro de Investigación en Biodiversidad y Conservación (CIByC), Universidad Autónoma del Estado de Morelos, Av. Universidad 1001 Col. Chamilpa, C.P. 62209 Cuernavaca, Morelos Mexico; Current address: UNAM campus Morelia, Antigua Carretera a Pátzcuaro No. 8711, ExHacienda de San José de la Huerta, C.P. 58090, Morelia, Michoacán Mexico

**Keywords:** Agaves, Mescal, Non-timber forest products, Risk management, Sustainable management

## Abstract

**Background:**

Mescal production is the main economic activity associated to agaves in Mexico, which involves 53 species mostly harvested from forests. The increasing mescal demand has influenced risk in both agave populations and mescal production, but other social and ecological factors also intervene. We hypothesized that the greater the risk the greater the complexity of management responses; otherwise, the greater the probability of populations’ depletion. We analysed this hypothesis by examining the diversity of risk conditions and management practices of *Agave inaequidens* in the state of Michoacán, in central-western Mexico.

**Methods:**

We studied five communities of Michoacán, documenting through 41 semi-structured interviews the use forms, risk perception, number of agaves annually extracted, and the management practices. Using a matrix with social-ecological and technological data analyzed by PCA, we evaluated similarities of management contexts. A data matrix with information on risk of agave populations, and other about management practices were analysed also through CCA and PCA. The scores of the first principal components were considered as indexes of risk and management complexity, respectively. A regression analysis of these indexes evaluated their relation.

**Results:**

We recorded 34 different uses of *A. inaequidens*, the most important being mescal production (mentioned by 76.1 % of people interviewed). Nearly 12.5 % of people practice only gathering, but others mentioned the following practices: Selective let standing of agaves for seed production (20 %); *in situ* transplanting of saplings; seed propagation in nurseries and saplings transplanting to forest (10 %); suckers transplanting (7.5 %); seed dispersal in forests; banning (5 %); enhancing of agave growth by removing tree canopies (2.5 %); transplanting from the wild to live fences (45 %); intensive plantations (35 %). The highest vulnerability of agave populations was identified in communities where risk is not counteracted by management. In two communities we identified the highest risk (annual extraction from 4,353 to 6,557 agaves), but different actions counteracting such risk.

**Conclusions:**

Interchange of knowledge and management experiences developed by handlers is crucial for the regional conservation, recovering, and sustainable management of *A. inaequidens* populations.

## Background

Subsistence of Mesoamerican human peoples has been characterized by a strong relation with their neighboring natural resources and ecosystems. Evidence of such relation are the more than 6000 plants species that are currently used to satisfy a broad spectrum of needs [1]. Nearly 1000 of these plant species are managed with different practices and intensities, and some 200 have clear signs of domestication [1–3]. Plant management practices and their intensity are guided according to human purposes and needs, and according to socio-cultural and ecological contexts [2, 3]. Some practices are usually aimed to ensure or increase the availability of a particular resource that is scarce, and others to promote particularly attractive variants within populations [3, 4]. Similarly, some plant resources have low importance, while others are particularly relevant not only in people’s subsistence but also as elements of their cultural identity. Among the latter relevant species maize, beans, chili peppers and squashes can be mentioned as the more representative in Mesoamerica [2], but others, nearly 100 to 200 plant species play important cultural roles, among them agaves have a special place. Studying the factors that currently influence decisions of management and domestication is particularly important in an area that has been recognized as one of regions of the world with the earliest signs of domestication and agriculture [2, 5, 6]. In addition, since these processes are continual and on-going, understanding them allows establishing viable bases for technological innovation for sustainable management of natural resources and ecosystems, one of the main current challenges of humanity [2].

*Agave* species have been important edible plant resources for Mesoamerican peoples since prehistory [5–7], and currently are used to satisfy multiple needs such as food, construction materials, fibers, beverages, living fences, medicine, tools, and religious ceremonies, among others. The relationship between agaves and humans has been cardinal to Mesoamerican cultures for whom agaves were in the past represented as deities and currently continue being part of their worldviews [8–12]. Most of agave traditional uses remain alive, especially in rural communities, where they represent not only a complement to the household’s subsistence, but in some cases the main or the only source of monetary incomes [5, 6]. The current activity that represents by far the main source of incomes from agaves is the traditional production of the distilled spirits called mescal, which is carried out by peoples of 24 (from a total 32) Mexican states and involves at least 53 agave species [11, 12]. The majority of these species are harvested directly from wild populations and there is scarce information about the existence or not of practices aimed to prevent their depletion. According to [12, 13], some few species are *in situ* managed and some others, even fewer, are cultivated *ex situ* with clear signs of domestication [7, 8].

During the last decades, regional, national and international demand of mescal spirits has pronouncedly increased, a phenomenon called the “mescal boom”. Currently, only eight states of Mexico have been recognized in the mescal’s appellation of origin (“Denominación de Origen Mezcal”, DOM, for its acronym in Spanish), among them the state of Michoacán, in which 29 municipalities received their recognition as part of the DOM in 2012. This fact increased the regional, national and international demand of Michoacán’s mescal, which has influenced an increasing pressure on wild populations of several species as well as on the few agave crop species used for this activity.

According to Gallardo *et al*. [14], the mescal production in Michoacán has a tradition of about 400 years old. Five *Agave* species are used for such purpose: *Agave tequilana* Web. and *A. americana* L. var. *subtilis* (Trel.) Valenz.-Zap. & Nabhan, which are domesticated species and exist only under cultivation, as well as *A. angustifolia* Haw., *A. cupreata* Trel. & Ber., and *A. inaequidens* ssp. *inaequidens* Koch. These species grow wild in the regional forests, although some communities have started to cultivate them in the last 20–25 years. Local governmental programs enhance mescal production and contribute to make it a growing activity, promoting its commercialization and exportation. However, they have forgotten the need to assure a sustainable provision of the mescal raw matter. The traditional mescal producers led by the market demand, have exceeded their traditional small production capacities, causing over-extraction of their wild and cultivated resources, as well as of other essential materials necessary for mescal production, particularly fuelwood and spring water. Governmental programs do not take into account neither issues of resources supply, programs and regulations of management of wild agave populations, nor the establishment of agaves and woody species plantations. This situation seriously endangers the wild populations of agave, the forests they form part, and the sustainability of this important socioeconomic activity. Therefore, social-ecological studies for establishing the bases of a long-term sustainable production are a priority in Michoacán and other areas of Mexico.

Some studies have documented cases of local extinction and endangered populations of wild *Agave* species related to the lack of management strategies in territories of mescal producing communities. These are for instance the cases of *Agave potatorum* Zucc. in the Tehuacán Valley; and *Agave cupreata* in the Balsas River basin [9, 10, 12, 15–17]. These semelparous species reproduce only by seeds [8] and like all mescal agaves, they are harvested when mature, just before the flowering stalk starts to develop, thus cancelling their only chance of sexual reproduction. The sexual reproductive event may take place when agaves reach about eight to 20 years old, a period varying among species, varieties regions, soils, and shade. With some important exceptions, the apparently prevailing extraction pattern of wild mescal agaves in Mexico is to collect all the reproductive individuals existing in a site and wait for the younger agaves to start flowering for cutting them. This practice, which is even more common as the demand of mescal increases in markets, modifies the natural demographic cycle of agaves thus surpassing the recovery capacity of populations.

As we examined previously in the case of *A. potatorum* [9, 10, 12, 15], the risk to local extinction of wild agave populations is determined by a complex relationship between distribution, abundance, reproduction type, length of life cycle, the intensity of extraction, which is in turn related to the demand of mescal in markets. But importantly, the risk also depends on the occurrence or not of practices directed to prevent the impact on agave populations. The risk should be visualized in two important dimensions, one is the probability of losing the populations of plant resources, and the other, the consequent probability of losing the socio-cultural and economic activity that mescal production represents for people.

Management may include regulations, planning, and actions directed to maintain populations and recover those decimated or extinct [5, 6, 8, 12]. As Gonzales-Insuasti *et al.* [18, 19], Arellanes *et al.* [20] and Blancas *et al.* [3, 13] documented, management responses are influenced by multiple factors related with socio-cultural, economic, ecological and availability pressures. Considering these studies, we hypothesized that similarly as in other species that are non-timber forest resources, in *A. inaequidens* we would find that the greater the risk the greater the management responses by people; but, alternatively, in situations in which no management is practiced, the greater the risk the greater the probability of agave populations depletion and eventually local extinction. Unfortunately, the latter situation is probably the most common and its characterization could help to identify the priority areas for protection, conservation and restoration activities, in order to construct sustainable forms of managing these plant resources.

Particularly important in this study was documenting the management strategies that are being designed and constructed by local people to decrease the risk of *A. inaequidens*. These strategies involve expressions of traditional ecological knowledge (TEK) on the target species and the ecosystems it belongs. In addition, the management techniques available may potentially help to implement them in the critical areas where the species is in high risk of disappearing. The different experiences of management practices are also expressions of different social and ecological contexts in which the risk of agaves and the management responses occur. Comparing those situations may allow understanding the context in which management techniques are being constructed, which in turn would help to provide criteria for planning innovation in the multiple contexts in which *A. inaequidens* is used to produce mescal. Interchange of experiences of management strategies between communities and agave handlers is, therefore, an attempt to construct a catalog of technical alternatives for a more rapid response to a problem that, because of the rhythms of markets, is now surpassing the rhythms of technical innovation in traditional contexts. Our study aims to contribute to document local management strategies and to develop proposals for research, actions, and responsibilities to implement by coordinated actions among producers, government, NGOs, and the academic sector.

## Materials and methods

### Study sites

**Fig. 1:**
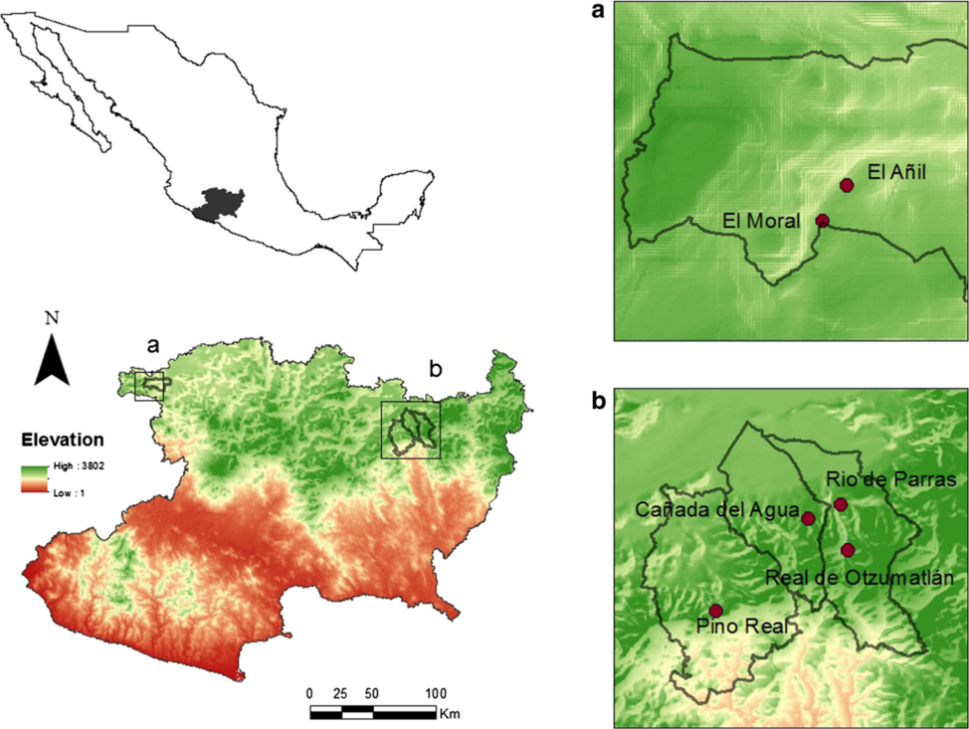
Study area. Location of communities and their municipalities: **a** The two localities of Barranca del Aguacate, Sahuayo, **b** (from left to right) Pino real, Charo, Cañada del Agua, Indaparapeo; Real de Otzumatlán and Río de Parras, Queréndaro, located in the Trans-volcanic Belt in the north of Michoacán

We studied five communities at the north of the state of Michoacán, Mexico (Fig. 1). Four of them are famous for mescal production, and the fifth is a community not producing mescal but that makes use of *A. inaequidens* as food and for other purposes (Table 1). The environment in the communities studied is mainly temperate (annual mean temperature being 17.7 °C, annual precipitation averaging 734 mm), and the prevailing economy is based on agro-pastoral activities. These communities are located in the Trans-Mexican Neo-volcanic Belt, which is the natural distribution area of the agave species studied [4].

**Table 1:** Study sites, ecological and economic aspects and number of mescal producers interviewed

Community	Municipality	Ecological and economic characteristics of the community area	Number of persons interviewed
Río de Parras	Queréndaro	Scrubland/rural: Agriculture (maize, beans and squash), cattle raising, mescal production.	10
Real de Otzumatlán	Queréndaro	Mountainous/rural: Agriculture (apple, plum and peach orchards), mescal production.	7
Cañada del Agua	Indaparapeo	Mountainous/rural: Agriculture (maize, beans and squash), mescal production.	7
Barranca del Aguacate (El Moral y el Añil)	Sahuayo	Scrubland/rural: Agriculture (maize, beans and squash; pitaya and hog plum orchards), cattle raising, gathering of wild resources, mescal production.	6
Pino Real	Charo	Mountainous/rural: Agriculture (maize, beans and squash; plum and peach orchards), timber industry, pine resin extraction.	11
Total	4	-	41

### Species studied

*A. inaequidens* ssp. *inaequidens* is regionally called “maguey alto”, “maguey bruto” or “mescal bruto”. It is a medium to large size rosetophylous plant producing 75 to 150 leaves broadly to narrowly lanceolate to oblanceolate, thick-fleshy, light green to yellow green. The leaves margin is undulate to repand and crenate; teeth are dimorphic, hence the specific epithet (*inaequidens* meaning unequal teeth in Latin), castaneous to dark brown; panicles 5–12 m tall, averaging 30 compact umbels. It is a monocarpic species that, according to mescal producers, reaches the reproductive stage after 10–20 years, depending on the quality of the site where it grows, mainly soils and shade. Before leaving their existence, these agaves produce one massive inflorescence and reproduces mainly from seeds. *A. inaequidens* may have vegetative reproduction in form of axil suckers, but we have observed it only as response to damage to the central meristem. It is pollinated mainly by bats, but birds and insects may also have an important contribution to its sexual reproduction [21]. Our preliminary studies on demographic patterns and on natural establishment of this species suggest that only approximately 1 % of the total seeds produced per reproductive individual become saplings after one year. *A. inaequidens* grows mainly in oak and pine-oak forests, and the populations’ spatial distribution have a clumped pattern called “magueyera” by the mescal producers. Its geographical distribution comprises mainly temperate zones of the Trans-Mexican Neo-volcanic Belt with elevations ranging between 1850 and 2480 m, in the Mexican states of Colima, Jalisco, Nayarit, Michoacán, México, Morelos and Hidalgo [8].

### Ethnobotanical information

This study analyzed the different forms of use, extraction and management of *A. inaequidens* ssp. *inaequidens* and documented the perception of risk of wild populations by local people of the communities studied (Table 1). After obtaining permission by the local authorities and people interviewed for carrying out our study, we conducted 41 semi-structured interviews from August 2011 to May 2013. Interviews focused on documenting social aspects that influence and define the skills and expertise of the agave handlers, such as age, education, migration, and transmission of knowledge. Additionally, we documented the uses of this species and evaluated the extraction rates, productivity, the relation of extraction rates and the number of sites of collection, type of extraction tools, among other aspects. We also obtained information on the traditional management techniques *sensu* Blancas *et al*. [3, 13], amounts of hand labour as the number of people that are involved in each management techniques, the number and complexity of management tools, and use or not of agrochemicals inputs, to diagnose the management techniques practiced by the mescal production units. In addition, we documented the social organization or partnership and regulations about the use of agaves and economic information about the price and the demand of mescal in the markets. The perception of risk was categorized considering all the responses obtained from the mescal producers, whether or not people identify differences in agave abundance in the last 30 years, how strong are those differences and if that view is accompanied by a negative or positive view of the populations’ perspective (Table 2). In addition, we considered their view on the harmful practices that cause damage to the environment and resources, and the role of management to decrease risk.

**Table 2:** Categories of perception of risk about changes in populations of *Agave inaequidens* subsp. *inaequidens* managed in the last 30 years. The column reporting the percentage of mention includes all people interviewed in this study. Particular records per agave handler are shown in Table 7

Category of risk perception	% of mention	Risk category
No change in relation to the past, positive perspective	5.26 %	1
Intermediate change positive perspective	10.53 %	2
Intermediate change	60.53 %	3
Intermediate change, negative perspective	5.26 %	4
Drastic, critical change	18.42 %	5
Drastic, critical change, negative perspective	7.89 %	6

### Extraction

In order to estimate the annual extraction of agaves for mescal production per community we inventoried the number of mescal production units in each community, the number of mescal batches produced per year per production unit, and the amount of agaves per batch.

### Data analyses

A database was constructed in order to systematize information on social and cultural aspects of mescal household’s handler’s, agave uses, mescal demand, management complexity (Table 3), and variables associated to risk (Table 4). We assigned qualitative values to these variables in order to identify their increasing complexity and risk, respectively. For the management practices documented in the field, we characterized their complexity and assigned to them values from 1 to 12, each number representing from lower to higher complexity. The relationship of the type and number of management practices was calculated by dividing the sum of the complexity value of the practices recorded by the number of practices recorded for each agave handler.

**Table 3:** Indicators considered for evaluating risk. Note that the categorical values are qualitative weights of each aspect, increasing according to their role determining risk

Factor	Criterion	Value
Handler age	>70 years	1
	60 to 70 years	2
	40 to 59 years	3
	20 to 39 years	4
Education	Technical level	1
	Secondary	2
	Primary	3
	None	4
Migration	No	1
	Yes	2
Knowledge transmission	Family/relative	1
	Employer	2
Productivity	0	1
	1–49	2
	50–99	3
	100–299	4
	300–599	5
	>600	6
Perception of risk	No difference	1
	Medium positive	2
	Medium	3
	Medium negative	4
	Critic	5
	Critic negative	6
Belonging to an organization	Yes	1
	No	2
Mescal market demand	Null	1
	Low	2
	Intermediate	3
	High	4
Price	High	1
	Low	2
Uses	One use	1
	Two uses	2
	Three uses	3
	Four uses	4
	Five uses	5
Harmful practices	None	1
	Medium	2
	High	3

**Table 4:** Indicators considered for evaluating the management complexity index

Factor	Criterion	Value
Extraction rate	0	1
	1–460 individuals	2
	460–920	3
	920–1380	4
	1380–1840	5
	1840–2300 individuals	6
Productivity	0	1
	1–49	2
	50–99	3
	100–299	4
	300–599	5
	>600	6
Extraction and management tools	One tool	1
	Two to three tools	2
	Four to five tools	3
	Six to seven tools	4
Practices weight sum/# practices	Recollection only to	
	different combinations of practices	From 1 to 9.75
Hand labour	Persons involved	From 2 to 19
Inputs (agrochemicals)	None	1
	One	1.25
	Two	1.50
	Three	1.75

With information on the ecological risk parameters, the management complexity and the social and economic pressures on agave populations, we conducted a PCA to evaluate general similarities and differences of social-ecological contexts and management strategies among handlers. In addition, we constructed two data matrixes with specific variables. One of them summarizing data about intensity and complexity of the management practices: hand labour, inputs used, number and type of management practices and extraction and management tools used (Table 3). The other matrix summarizing information about risk of agave populations that manage each handler, which includes information about the handler characteristics, such as age, education, among other aspects. We assigned higher risk value to younger agave handlers since they have the capacity of extracting more agaves, but also because over time the handlers adjust and define the better way to conduct a management practice; therefore, more experience is accumulated by the handler and there is a direct relation between age and experience. We also assigned a higher risk value to less educated agave handlers since in the overall strategy, i.e. the ability of the handler to deal with changes that are generated from external factors such as changes in demand, regulations of markets, or legal situations that can affect the performance of their activity. We in addition included indicators like productivity, risk perception according to the annual availability of resources and rhythm of extraction (Table 4). We performed two additional PCAs, one for analyzing the risk associated to the contexts of extraction of each mescal producer, and the other for analyzing the management complexity and intensity. The scores of the first principal components were considered as indexes of risk and management complexity, respectively [3, 9]. A regression analysis was conducted to explore the relationship between these two indexes. In addition, through the programme R [22] we performed a Canonical Correspondence Analysis (CCA) to estimate how much the variation of data about management are explained by variables of risk. The model used was based on that developed by Borcard *et al.* [23] which makes use of a response matrix Y (in this case the management matrix) and a matrix X with explanatory data (variables of risk). Through ANOVA we then identified which risk variables more significantly contribute to explain their effect on management.

## Results

### Uses of *Agave inaequidens* ssp. *inaequidens*

**Fig. 2:**
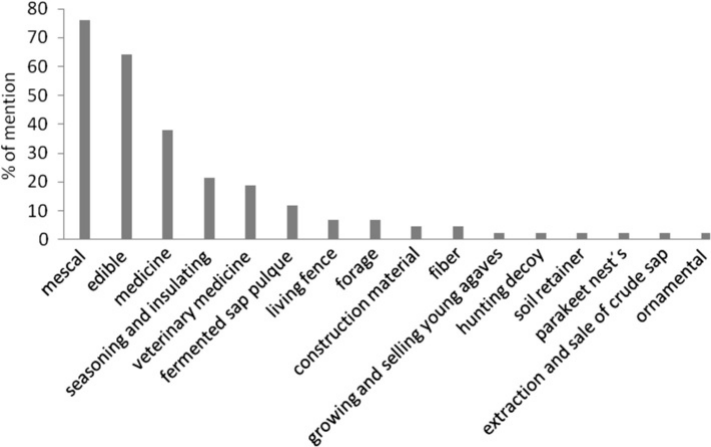
Documented use categories of *Agave inaequidens* by the agave handlers and percentage of mention in the 41 interviews conducted in the study area

We documented 16 use categories and 34 specific uses of *A. inaequidens* in the study area (Table 5, Fig. 2). The following are the use categories recorded in order of importance according to their percentage of mention by people: Mescal production (76.1 %), food (64.2 %), medicine (38 %), seasoning and insulating (21.4 %), veterinary (19 %), fermented sap or “pulque” production (11.9 %), live fences, fodder (7.1 %), materials for construction and fiber (4.7 %), growing of young agaves for commercialization, hunting decoy, barrier for preventing soil erosion, nests for parakeet and bird pets, and ornamental purposes (2.3 %).

**Table 5:** Use categories and specific uses of *Agave inaequidens* subsp. *inaequidens* documented in this study (ordered according to the percentage of mention)

Use category	Specific use	Usedpart
Distilled beverage	Mescal	Stem and leaf bases
Food	Sweetfood	Tender floweringstalk
	Sweetfood	Stem and leaf bases
	“Aguamiel”	Stemsap
	Food	Flowerbuttons
	“Nixtamal” tortilla dough complement	Stem
Medicine	Gastro-intestinal discomfort	Sap
	Internal and externalbruises	Leaf
	Respiratory illnesses	Sap
	Fractures	Leaf
	Articulation pain	Sap
	Rheumatism	Leaf
	Pressure regulation	Sap
	Gastritis	Sap
	Ulcer	Leaf
Seasoning and insulating	“Barbacoa”	Leaf
Veterinary medicine	Parasite control	Leaf
	Internal and external bruises	Leaf
	Fractures	Leaf
	Fatigue releaf	Sap
Fermented beverage	“Pulque”	Sap
Living fence	Exclusion of cattle	Complete living plant
	Delimiting property	Complete living plant
Forage	Cattle fodder	Leaf
Construction material	Door fence	Floweringstalk
	Water pipe	Floweringstalk
	Roof gutter	Floweringstalk
	Pole	Floweringstalk
Fiber	Textil confection	Leaf
Plant sale and trade	Growing and selling young agaves	Seed
Hunting	Hunting decoy	Inflorescence
Soil retainer	Erosion control	Complete living plant
Parakeets’ nest	Parakeets’ nest confection	Flowering stalk
Extraction and sale of sap	Sale of extracted sap	Leaf
Ornamental	Garden ornamental	Complete living plant

### Extraction rates

The extraction of agaves for mescal production is the main activity in all the communities studied, except in Pino Real, where the main use of *A. inaequidens* is the consumption of its tender inflorescence as sweet food. The extraction of agaves for preparing mescal is carried out using a sharp tool called “trinchera”, a kind of thin axe with a large wooden handle ending in a hook, which is used to cut the leaves and remove them. Axes and machetes are also used to cut the bases of agave stems, and more recently some mescal producers use chainsaws to do this latter step. The resulting raw material for mescal production is the stem and leaf bases, that are locally called “cabezas” (heads), or “piñas” (“pineapples”, because of its resemblance to the infrutescences of those plants).

Information on the number of mescal batches per year per householder, the number of agaves extracted per batch and the estimation of agaves extracted per year per community, is summarized in Table 6. The highest extraction in a year was recorded in Otzumatlán (6,557 agaves); on average, a mescal producer carry out 7.7 ± 7 batches per year, and in total 54 batches per year in the whole community. A batch is composed by 121.4 ± 63 agaves and on average, mescal producers extract 749.2 ± 609 agaves per year. In Parras, mescal producers prepare 3.3 ± 2 batches per year (in total 27 batches), each batch composed by 173.7 ± 114 agaves. Mescal producers extract 542.5 ± 395 agaves per year, and the whole community extracts 4,353 agaves per year. In Cañada del Agua, mescal producers extract on average 61.5 ± 41 agaves and the extraction totalizes 1,845 agaves yearly; mescal producers prepare 3.7 ± 2 batches per year (in total 30 batches) composed by 61.5 ± 41 agaves. In Barranca del Aguacate, mescal producers make on average 2.5 ± 1 batches per year (15 batches in the community), a batch composed of 19.5 ± 6 agaves. In this community, the mescal producers extract on average 49 ± 33 per year, the annual extraction is in total 292 agaves. In Pino Real, people extract 67 inflorescences per year and on average a householder cut 6.09 ± 6.7 inflorescences.

**Table 6:** Extraction amounts and averages of batches per year, agaves extracted per batch and the estimation of agaves extracted per year per production unit/householder

Community	Production unit/householder	Average batches per year	Agaves per batch	Agaves per year
Real de Otzumatlán	RO2	1	100	100
	RO6	2	100	200
	RO7	2	180	360
	RO1	5	240	1200
	RO4	8	80	640
	RO3	13.5	70	945
	RO5	22.5	80	1800
	*average*	7.7 ± 7	121.4 ± 63	749.2 ± 609
Río de Parras	RP9	1	120	120
	RP5	2	90	180
	RP6	2	400	800
	RP8	2	100	200
	RP3	3	80	240
	RP7	5	150	750
	RP2	7	150	1050
	RP1	5	300	1000
	*average*	3.3 ± 2	173.7 ± 114	542.5 ± 395
Cañada del Agua	CA1	3	30	90
	CA2	3	150	450
	CA3	3	28	84
	CA4	8	70	560
	CA5	2	48	96
	CA6	4	40	160
	CA7	6	90	540
	CA8	1	36	36
	*average*	3.7 ± 2	61.5 ± 41	252 ± 223
Barranca del Aguacate (El Moral y El Añil)	BA1	4	25	100
	BA2	5	14	70
	BA3	1	15	15
	BA4	2	25	50
	BA5	2	25	50
	BA6	1	13	13
	*average*	2.5 ± 1	19.5 ± 6	49 ± 33
Pino Real	PR1			18
	PR2			10
	PR3			0
	PR4			9
	PR5			16
	PR6			4
	PR7			1
	PR8			9
	PR9			0
	PR10			0
	PR11			0
	*average*			6.09 ± 6.7

### Management techniques of *Agave inaequidens*

The following management techniques and the percentage of people interviewed mentioning to practice them were registered:

#### *In situ* management

1. Simple gathering. In total, 12.5 % of people interviewed mentioned to practice simple gathering, harvesting agaves from the forest without any other kind of intended management. This practice involves the removal of complete individuals and no replacement of plants is carried out. It is mainly conducted by people dedicated to extract agaves, but who are not owners of the land they use. They are paid for extracting agaves but they cannot make management decisions. They collect mature and nearly mature agaves, as well as “gelded” agaves (details about this practice are explained later in this section), gelded by the landowners with the purpose of selling them to the mescal producers. The number of people that participate in the extraction varies from three to six persons depending on the number of agaves they buy. They work during two to three days, one or two days cutting agaves and removing their leaves, and one more to loading and transporting them to the mescal production unit. Depending on how far is located the land of agave extraction they make use of donkeys or mules for the nearest places, and trucks for further away places. Depending on the number of agaves extracted, they make several trips to transport the entire load or decide to pay the service of big trucks to do the work.
2. “Castration” or “gelding”. People use to cut the tender inflorescences when these start growing (45 % of people mentioned to carry out this practice). This practice is called “castration” or “gelding” by the mescal producers since it cancels the development of the inflorescence of the only reproductive event. The practice has the purpose of maintaining or increasing the levels of fructanes contained in the agave stems. Agaves that were gelded are let standing for at least one year, in this way the fructanes become more concentrated in the stem and leaf bases. In agaves that are let standing more than one year, this practice may favor the production of asexual axillary or inflorescence bract suckers, a phenomenon occurring rarely in nature. Suckers can be transplanted by people.
3. Tolerance of seed producer agaves. This practice consists in left standing some mature agaves until seeds production (20 % of people interviewed said to carry out this practice). This practice maintains the natural recruiting of individuals of the population. It is conducted when the mescal producers are owners of the forestland that is being harvested, or when they have an agreement of extraction exclusivity with the landowner and, therefore, the right to manage the agave population. These agreements in some cases are historical and inherited. Some people prefer to let standing the small agaves, others said to let agaves of any size, whereas the minority said to let standing the largest sized agaves. On average, the proportion of agaves that they let standing to freely reproduce and disperse their seeds is one of 20 mature agaves (5 % of reproductive agaves).
4. Transplanting of juvenile agaves. Transplanting of juvenile agaves from undesirable to favorable places in forest is practiced by 10 % of people interviewed; mainly those that are landowners or have the agreement referred to above. This practice protects the younger agaves from density dependence factors and the damage caused by cattle. This activity is carried out when plantlets or juvenile agaves are in high densities in relatively small spaces; people know that in those circumstances, plantlets’ development is compromised because of the competition for resources. In addition, people use to transplant plantlets from cattle trails where plantlets are in danger of being trampled to safety sites.
5. Weeding. This action was mentioned by 10 % of people interviewed. It is conducted one or two times per year manually, mechanically, and chemically. The manual techniques is conducted with hoe or machete to cut the growing weeds; this weeding generally requires work of two to four persons, depending on the size of the managed land, and it takes one to three working days. The mechanical technique involves the use of gasoline string trimmer, and it requires approximately half the hand labor and half the time of the former type. The chemical technique involves the use of herbicides, and it is performed once per year. Only one person said to use it, requiring one working day.
6. Seed sowing in nurseries and transplanting of saplings into forest. Reforestation of plants grown up from seeds in nurseries was practiced by 10 % of people interviewed. This practice allows the germination and establishment of a higher percentage of individuals than the natural establishment. People that practice this management said that nearly 90 % of the seeds that are sown can germinate and reach the stage of sapling. One year old saplings are transplanted to wild populations during the rainy season (July - September), taking care of conducting the transplantation ensuring enough space between plants to prevent competition among themselves. This practice involves a variable number of people, but regularly is performed by the mescal producer and his family.
7. Transplanting of suckers. Transplantation of sucker derived from “castrated” and “aguamiel” sap-extracted individuals to save sites is practiced by 7.5 % of people interviewed. This practice allows that suckers naturally not reaching the substrate to do it. When the harvest of these individuals takes place, some of them may have bract or axillary suckers. Most mescal producers said they do not use to transplant these structures, since they consider them unviable to grow and survive. However, some persons take the time to transplant sucker to favorable safety sites where they are protected from the livestock trampling with successful results. In the case of individuals that their sap were extracted to obtain “aguamiel”, in which the main meristem was carved to obtain the sap, the individuals are left for about two to three years to let the axillary suckers growing and then people transplant them.
8. Seed collection and dispersal. Seed collection and dispersal in favorable places in forests is practiced by 5 % of people interviewed. This practice allows that a higher percentage of seedlings are established in comparison with the natural establishment. The seed collection occurs from March to May. The most common way to get seeds is to cut down the whole inflorescence and harvest only the mature capsules before releasing the seeds (commonly at the stage of yellow and brown color), the empty and immature capsules are let there. Later on, during the rainy season, seeds are separated from capsules and stored in bags, and then dispersed in favorable sites (terrains partially without grass and herbs, and with adequate moist conditions). Presence of rocks and mosses is particularly important for the establishment of agave plantlets. This action can be carried out by a single person in one day. Another way in which this practice is carried out is cutting the whole inflorescence, transporting it and raising or establishing it in the favorable place letting the mature capsules to disperse their seeds and to let the immature ripping.
9. Biannual banning. Nearly 5 % of people interviewed said to practice a kind of banning, let some wild populations recovering by not extracting agaves for at least two years. This practice allows that mature individuals blooms, disperse seeds and establish naturally, contributing to a good demographic performance of the population. This decision is made depending on the availability of agaves from other sources where they can buy the raw material and let their own populations to recover. For instance, we recorded a forestland that has been recovering for ten years.
10. Enhancing agave growth by removing forest canopy. Cutting down forest cover to enhance agave populations is practiced by 2.5 % of people interviewed. This practice allows a better development of individuals of the population and the mescal producers prefer agave individuals that are growing at higher sun exposures because of their higher levels of carbohydrates. It is performed by cutting down all the trees and bushes surrounding and shading a wild agave population to enhance its development, by eliminating competition and increasing insolation. They use chainsaw and “machetes” for conducting this activity.

#### *Ex situ* management

11. Transplanting. Transplanting of juvenile plants from wild populations to transformed areas, mainly orchards and living fences in agricultural plots was said to be practiced by 45 % of people interviewed. This practice is carried out before the rainy season to ensure or making more probable the plant establishment in human transformed areas and ensures the resource availability. It is carried out sporadically when the mescal producers are out in the field doing other activities; in that context they collect juvenile plants and bring them to home to include them as part of live fences of agricultural plots or home gardens.
12. Cultivation in plantations. Intensive cultivation was mentioned to be practiced by 35 % of the people interviewed. This practice ensures the resource availability. This kind of management is characterized by having a high transformation level, the agaves are planted in rows and agrochemicals are invariably used, mainly for weeding and control of insect and fungal agave pests.
13. Seed sowing. Seed collection and plantlets growing in nurseries for later transplantation, selling or bartering was mentioned to be carried out by 25 % of people interviewed. People that practice this management says that nearly 90 % of the seeds that are sown can germinate and became sapling. As described earlier, seeds are collected from wild populations and planted in seedbeds delimitated with wooden boards. People use to put a layer with cattle dung at the bottom, and an upper layer with sand, where seeds germinate. Seeds and plantlets are irrigated once per week and weeded twice per month let them growing for at least one year. Then plantlets are transplanted, commercialized or bartered.

### Perception of risk

Based on the testimony of all 41 agave handlers interviewed, we classified their views on risk of agave populations and their perspectives, into six categories. In order of importance (according to their percentage of mention) (Table 2), these categories are: (1) Intermediate change (60.53 % of mescal handlers) the perception is that nowadays abundance of wild populations has decreased approximately to one half compared with their abundance 30 years ago; (2) drastic critical change (18.42 %), the perception is that wild populations are very scarce; (3) intermediate chance with a positive perspective (10.53 %), current abundance of wild populations is nearly one half of what they were, but people is carrying out management actions; (4) drastic critical change with a negative perspective (7.89 %), wild populations are very scarce and their depletion is progressive because of the absence of management; (5) intermediate change with a negative perspective (5.26 %), current abundance of wild populations is nearly one half of what it was and that their depletion is progressive because of the absence of management; (6) no change in relation to the past with a positive perspective (5.26 %), wild populations are as abundant as they were 30 years ago and that management actions are being carried out to maintain that abundance. One particularly important aspect mentioned by the majority of the agave handlers interviewed is that nowadays the size of mature agaves is smaller than 30 years ago.

### Classification of mescal producers’ strategies

**Fig. 3:**
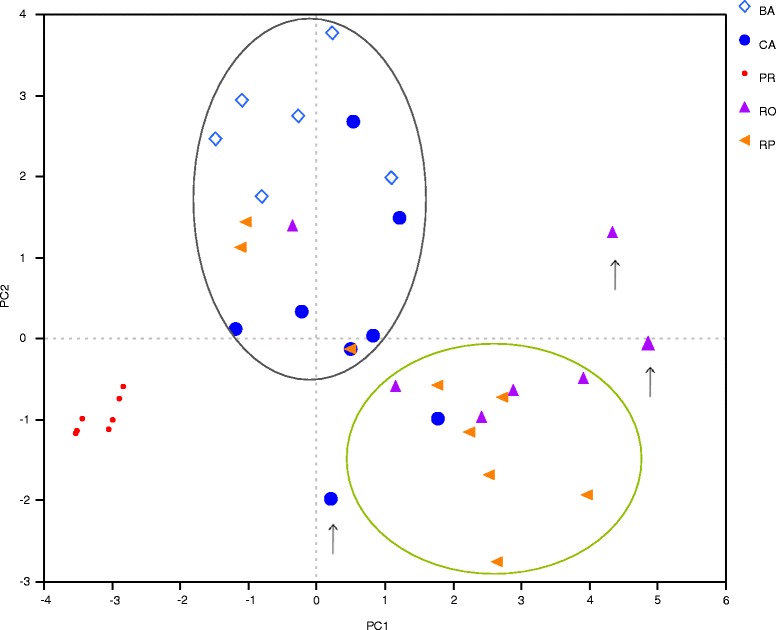
Spatial arrangement of handlers’ strategies according to the PCA performed. BA = Barranca del Aguacate, CA = Cañada del Agua, RO = Real de Otzumatlán, RP = Río de Parras, and PR = Pino Real. The gray oval groups the second group and the green oval groups the third group. Arrows points the out layer handlers recognized in their communities for having a successful management

Through PCA (Table 7, Fig. 3) of information about agave management strategies, we identified three main groups of mescal producers. The one of Pino Real, which is clearly differentiated and characterized by low hand labour employed in the scarce management practices (Fig. 3, red dots). They practice only simple gathering; their interaction is focused on the use of inflorescences as food, they are not mescal producers. The second group includes mescal producers from Barranca del Aguacate, Cañada del Agua and some few producers from the other communities, who highly migrate and have a negative perception about the future of *A. inaequidens* populations (Fig. 3, gray circle). The third group includes people from Parras and Otzumatlán, which extract the highest amounts of agave recorded, employ high hand labour, have a diversified management of agave populations and make use of tools for the extraction and management of agaves (Fig. 3, green circle). Some on the agave handlers included within this group are part of an organization of mescal producers, and their principal benefits depend on intensifying the production and commercialization of mescal and partnership doesn’t have a great influence on the management complexity. The out-layers are recognized as successful agave handlers and mescal producers in their communities. Their mescal has high demand in the local market, they have high productivity as well, and their management practices are the most diversified and effective (Fig. 3, arrow pointed icons).

**Table 7:** Information about socio-cultural and management aspects of the handlers’ strategies, management complexity and risk indexes recorded in the north of Michoacán. BA = Barranca del Aguacate, CA = Cañada del Agua, RO = Real de Otzumatlán, RP = Río de Parras, PR = Pino Real

Handler	Age	Education	Migration	Uses	Transmission	Productivity	Perception	Partnership	Harmful practices	Mescal demand	Price	Extraction rate	Extraction/management tools	Weight sum/# practices	Hand labor	Imputs
BA1	2	4	2	6	2	4	6	2	1	3	3	2	2	4.33	7	1
BA2	3	4	2	4	1	5	5	2	1	4	3	2	3	8	8	1.25
BA3	3	4	2	2	2	5	6	2	1	2	3	2	3	6.5	6	1
BA4	2	4	2	3	2	4	5	2	1	2	3	2	1	1	4	1
BA5	3	4	1	3	2	5	5	2	1	2	3	2	2	5.5	6	1
BA6	3	4	2	1	2	5	5	2	1	2	3	2	1	1	4	1
CA1	3	4	1	3	1	5	2	2	1	4	3	2	3	5	8	1.25
CA2	3	4	2	5	1	3	5	2	1	4	3	2	2	5.5	6	1
CA3	4	1	1	3	1	5	1	2	1	3	2	2	3	4.5	8	1.25
CA4	3	4	1	4	1	3	3	2	1	4	2	3	3	4	8	1.25
CA5	3	4	1	3	2	4	3	2	1	2	2	2	3	7.33	8	1
CA6	1	4	1	2	1	4	3	2	1	3	2	2	2	1.5	5	1
CA7	3	4	2	5	1	3	2	2	1	4	3	3	3	6	6	1
CA8	2	3	1	5	1	5	3	2	2	3	2	2	5	8.5	11	1.25
RO1	3	3	2	3	2	2	3	1	2	3	2	4	4	8.4	9	1.5
RO2	2	3	2	2	1	4	5	2	1	3	2	2	2	4.33	7	1
RO3	3	4	2	5	1	2	2	2	2	4	3	4	7	6.66	11	1.75
RO4	3	3	1	3	1	3	3	2	1	3	2	3	4	7.33	8	1.25
RO5	3	4	2	1	1	1	3	2	1	4	3	6	5	6.125	19	1.25
RO6	2	4	1	2	1	2	4	2	2	3	2	2	6	7.75	9	1.5
RO7	2	4	2	2	2	3	3	1	3	3	2	2	4	5.2	10	1.25
RP1	3	4	1	3	1	4	3	1	2	4	2	2	5	8.75	10	1.25
RP2	3	3	1	4	1	3	3	1	2	4	2	4	6	8.4	9	1.5
RP3	3	3	2	2	2	4	4	1	2	2	2	2	5	8.6	9	1.5
RP4	3	4	2	1	2	6	3	2	2	3	2	1	6	9.75	8	1.5
RP5	2	3	1	2	1	4	2	1	2	3	2	2	5	9.5	8	1.5
RP6	2	2	2	1	2	3	1	2	1	3	2	3	3	1	4	1.25
RP7	2	4	1	2	1	2	3	2	2	3	2	3	4	8	8	1.5
RP8	3	2	2	2	1	3	6	2	1	2	2	2	2	1	4	1
RP9	2	4	1	4	1	3	5	2	1	3	2	2	2	1	4	1
PR1	2	4	1	4	1	5	3	2	1	1	1	2	1	1	2	1
PR2	4	4	1	2	1	5	3	2	1	1	1	2	1	1	2	1
PR3	2	4	1	1	1	6	3	2	1	1	1	1	1	1	2	1
PR4	3	4	1	2	1	5	3	2	1	1	1	2	1	1	2	1
PR5	2	4	1	3	1	5	3	2	1	1	1	2	1	1	2	1
PR6	4	4	1	2	1	5	3	2	1	1	1	2	1	1	2	1
PR7	3	4	1	2	1	5	3	2	1	1	1	2	1	1	2	1
PR8	3	4	1	2	1	5	3	2	1	1	1	2	1	1	2	1
PR9	3	4	1	2	1	6	3	2	1	1	1	1	1	1	2	1
PR10	3	4	1	3	1	6	3	2	1	1	1	1	1	1	2	1
PR11	3	4	1	3	1	6	3	2	1	1	1	1	1	1	2	1

The variables with higher weight in the classification of management patterns through the first principal component are: (1) Extraction and management tools employed; (2) labour hand employed; (3) the relation between the sum of practices’ weight and the number of management practices; (4) the number of inputs employed (agrochemicals); (5) the mescal demand in the regional market, and (6) productivity (Table 8). This principal component explains 38.06 % of the variation. In the second principal component (explaining 14.70 % of the variation), the following variables are the most relevant: (1) perception of risk and conservation, (2) the price of the product in the regional market, and (3) migration. In the third principal component (10.76 % of the variation), knowledge transmission is the main factor. The three principal components explain 63.52 % of the variation (Table 8).

**Table 8:** Eigenvectors of the first three Principal Component Analysis classifying the mescal handlers’ strategies of management in the communities studied

Factor	PC1	PC2	PC3
Age	−0.068282	−0.116065	−0.165699
Education	−0.224774	0.2203967	0.0037511
Migration	0.4067214	**0.6465633**	0.3079537
Uses	0.1999348	0.2767164	−0.435015
Transmission of experience	0.1841198	0.4162504	**0.7246147**
Productivity	**−0.723315**	−0.156627	0.2888761
Perception of risk	−0.122723	**0.7397652**	0.2680137
Partnership	−0.527898	0.3742939	−0.45823
Labour hand	**0.8964532**	0.0597666	−0.119926
Harmful practices	0.6838442	−0.400892	0.4411164
Mescal demand	**0.8270976**	0.2097866	−0.312528
Price of mescal	0.5993685	**0.6988236**	−0.066022
Extraction rate of agaves	0.6542544	0.0829236	−0.431739
Extraction/management tools	**0.9188682**	−0.226786	0.0378166
Weight sum/number of practices	**0.842084**	−0.093713	0.123967
Inputs	**0.8258588**	−0.383827	0.0787319

Values in bold have a higher weight in the classification of management strategies

### Risk and management intensity indexes

Table 9 shows the scores of the first components of the PCAs considered as the risk and management complexity indexes, respectively. The regression analysis (Fig. 3), indicates the highly significant linear relation between risk and management intensity indexes (R^2^ = 0.431, *P <* 0.0001). Handlers above the line are people investing complex management actions according to the risk of the populations they use. Contrarily, handlers below the line are people investing deficient management actions according to the risk of the populations they use. The distance of the position of points (agave handlers) with respect the line represent better (points above the line) or worst (points below the line) conditions of management counteracting the conditions of risk.

**Table 9:** Management complexity and Risk indexes estimated for the different agave handlers included in this study. BA = Barranca del Aguacate, CA = Cañada del Agua, RO = Real de Otzumatlán, RP = Río de Parras, PR = Pino Real

Locality/handler	Risk	Management intensity
BA1	1.02123	−0.32146
BA2	1.07315	0.63236
BA3	−0.37202	0.0321
BA4	−0.06862	−1.00375
BA5	−0.24649	−0.24262
BA6	−0.69915	−0.9907
CA1	0.80302	0.32126
CA2	1.51117	−0.26873
CA3	0.16667	0.26941
CA4	1.04751	0.14569
CA5	−0.23404	0.21586
CA6	0.04977	−0.72568
CA7	1.53666	−0.09162
CA8	0.85951	1.19185
RO1	0.46565	1.1037
RO2	0.21141	−0.32146
RO3	2.06374	1.88171
RO4	0.55319	0.66238
RO5	1.9043	1.15196
RO6	0.64681	1.46983
RO7	0.20677	0.59731
RP1	0.69189	1.14952
RP2	1.01149	1.45879
RP3	−0.39559	1.41307
RP4	−0.60963	1.72
RP5	0.11415	1.45119
RP6	−0.30082	−0.38622
RP7	0.59066	1.05276
RP8	−0.13592	−0.84579
RP9	0.52388	−0.84579
PR1	−0.83461	−1.1011
PR2	−1.32203	−1.1011
PR3	−1.63407	−1.0423
PR4	−1.28464	−1.1011
PR5	−1.04093	−1.1011
PR6	−1.32203	−1.1011
PR7	−1.28464	−1.1011
PR8	−1.28464	−1.1011
PR9	−1.46514	−1.0423
PR10	−1.25882	−1.0423
PR11	−1.25882	−1.0423

### Risk factors influencing management responses

**Fig. 4:**
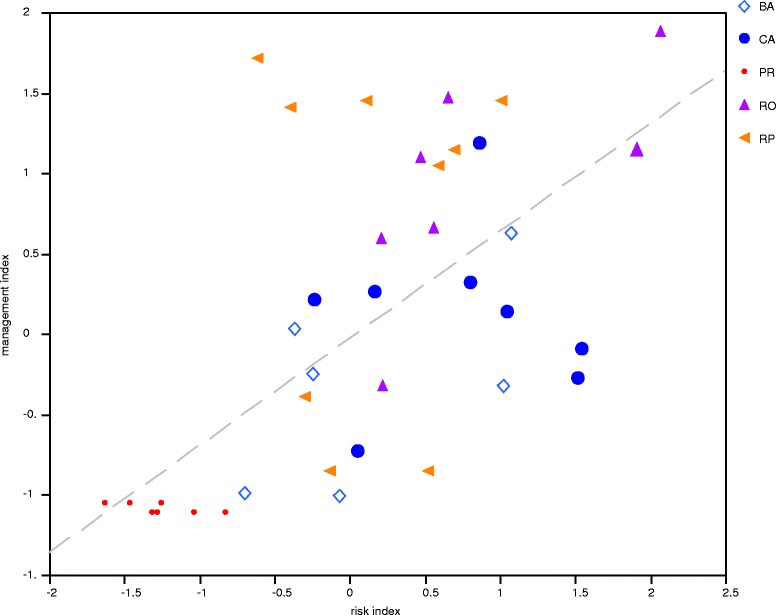
Regression analysis of the management complexity index as a function of the risk index calculated as the scores of the first principal component of PCAs (R^2^ = 0.435, *P <* 0.00). BA = Barranca del Aguacate, CA = Cañada del Agua, RO = Real de Otzumatlán, RP = Río de Parras, and PR = Pino Real. Mescal producers above and close to the dashed line are those with good management practices since their responses are adequate according to risk index. Those below the line indicate producers more vulnerable since their management strategies are not good enough than expected according to the risk representing their practices

**Fig. 5:**
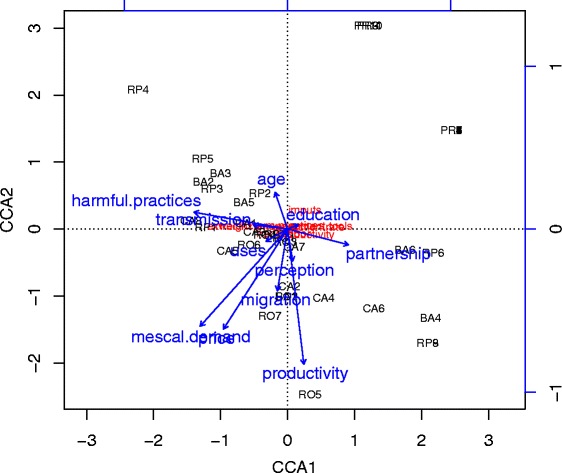
Plot of the CCA performed between the X matrix (risk variables for agave populations and mescal production) and Y matrix (variables of management complexity on *Agave inaequidens*). Showing how agave handlers (black) and management complexity (red) are influenced by risk variables (blue arrows),vectors length indicate the contribution of risk variables for explaining the management patterns practiced by agave handlers from BA = Barranca del Aguacate, CA = Cañada del Agua, RO = Real de Otzumatlán, RP = Río de Parras, and PR = Pino Real

The CCA indicates that variation in the form of agave management is 60.54 % explained by the risk variables. According to ANOVA, a highly significant influence was identified in the following variables: the demand that their mescal has in markets and the implementation or not of harmful practices in agave management, followed by a significant influence of partnership associated to the practices of mescal production, the productivity and knowledge transmission (Table 10, Fig. 4 and Fig. 5).

**Table 10:** ANOVA associated to the CCA of the matrix X (risk variables) and matrix Y (management variables). Number of permutations: 999

Risk variable	Df	Chisq	F	P(>F)
Age	1	0.0015	1.1958	0.294
Education	1	0.0005	0.4124	0.739
Migration	1	0.0014	1.1748	0.257
Uses	1	0.0013	1.0656	0.326
Transmission	1	0.0032	3.1688	0.076 ∙
Productivity	1	0.0041	3.2399	0.037*
Perception	1	0.0018	1.4562	0.178
Partnership	1	0.0057	4.5223	0.013*
Harmful practices	1	0.0126	9.9879	0.001***
Mescal demand	1	0.0218	17.2152	0.001***
Price	1	0.0021	1.6753	0.194
Residual	29	0.0368		

Significance: ∙*P < 0.05, ** *P <* 0.01, ***P < 0.001

## Discussion

### Uses

In Michoacán, *Agave inaequidens* is the agave species with the highest number of use categories documented in the literature. It is followed by *A. marmorata* with 14 use categories and *A. potatorum* with 12 registered by Delgado–Lemus *et al*. [9] in the Tehuacán Valley. It may be expected that other wild species that grow near these biocultural regions have similar number of use categories, since most agaves species have a noteworthy relation with human cultures all over the Mexican territory as Gentry [4] and Colunga-GarcíaMarín [24, 25] have documented.

*Agave inaequidens* represents a plant resource highly valued by the rural communities of Michoacán, and nowadays represents a multiple source of benefits and potential uses that are falling into disuse. This is for instance the making of ropes that are currently used in the neighboring state of Jalisco [26]. The decay of use of agave fibers has been documented for several species that were relevant since pre-Columbian times but that since the second half of 20th Century sharply declined because of the development and introduction of synthetic fibers. These are for instance the cases of *A. fourcroydes* and *A. lechuguilla* [25, 27]. In the case of *A. inaequidens*, the promissory markets for fiber extraction in Michoacán needs further ecological, technological and socio-economic studies.

### Extraction

Extraction of agaves for mescal production and extraction of tender inflorescences for food are undoubtedly the uses determining the higher impact on agave wild populations, because of the number of individuals extracted, and because these practices cancel their only sexual reproductive event. León [21] reported that the mean number of fruits and seeds per fruit are 2304 ± 547.58 SE and 375 ± 56.67 SE per individual plant of this species in the study area, respectively. This information allows estimating 805,520 seeds produced on average by one single rosette of *A. inaequidens*. Therefore, the extraction of agaves in Otzumatlán cancels the annual production of nearly 5.2 billion seeds, in Parras 3.5 billion, in Cañada del Agua 1.4 billion, and in Barranca del Aguacate 235 million. Our current studies on population ecology and demography with *A. inaequidens* identified that sexual reproduction is the main source of recruitment, and like documented in other *Agave* species [12, 28–33], and according to our preliminary results, this species is characterized by presenting low recruiting rates, nearly 1 % of the seeds become saplings after one year. The loss of this high number of possible seedling therefore must have a major impact on the wild population dynamics, especially in those where management practices were absent.

### People’s perception of risk

Considering the cultural importance of this multipurpose resource and the significant incomes it generates for household economy, it is important to understand the local people’s worries about the threats on the resource and the economic activity. We found a gradient of perceptions related with needs of management perceived by people for ensuring the conservation of this agave species, considering the past and present conditions and the future perspectives. Nearly 20 % of people affirmed that there has been a drastic change, and if no management actions are carried out the agave is in real danger. The majority of agave handlers said that the abundance of wild populations has decreased but most people have a positive perspective. The majority agrees that in the past the sizes of the mature individuals were massive and that their extractive efforts were focused on these sizes, the majority of agave handlers let the small agaves to reproduce, which suggests that this artificial selection has favored the reproduction and higher frequency of small size agaves in the forests. This process, according to the handlers’ testimony, has led to a historical decrease in the size of mature agaves in wild populations as has been evaluated and reported by Figueredo *et al*. [34]. The most common perception of people is a testimony that in general the populations are declining in both quantity and quality.

### Management strategies

Our model analyzed socio-ecological and technological variables influencing differences in how people practice management of one single plant species that represents a highly appreciated resource. The use and management of this particular resource is only a fraction of several general subsistence strategies carried out by people of the studied communities, since strategies involve other socio-cultural activities such as agriculture, orchards harvesting, timber harvesting, gathering of other NTFPs, and pastoral activities. Incomes derived from the mescal production and commercialization is a complement to the household economy and how important is that complement may be related to the management strategies that different households carry on. Strategies of agave the handlers documented in this study, conform a gradient of management complexity. This gradient is determined by an intricate relation of multiple factors of distinct nature. We identified that factors that have a mayor contribution on the classification of the agave handlers is directly related to the implementation of technologies such as modern tools like the chainsaw, the quantity of inputs and the quantity of labour hand employed, the support of the implementation of different management practices and different combinations of them.

The grouping pattern of agave handlers’ strategies in Fig. 3 and the risk and management indexes regression in Fig. 3 are generally consistent. The first group, at the bottom of the management complexity gradient, is clearly formed by the agave handlers of Pino Real, the less vulnerable since they are not mescal producers and the extraction rate of agaves is very low compared with those of the other communities and they do not obtain monetary incomes from this practice. People of Pino Real do not require practicing complex management forms or using specialized tools to satisfy their need of this agave products. The second group, at the middle of the gradient, is mainly formed by agave handlers from Barranca del Aguacate and Cañada del Agua, where mescal production is one important source of monetary income. This group is the most vulnerable, although their extraction levels are intermediate, they make use of specialized tools to extract agaves for mescal production and pay labor hand to carry out their few management practices. In Barranca del Aguacate, according to the testimony of the interviewed people, the extraction has determined local extinctions directly related to the lack of management practices. The third group, at the top of the gradient, is mainly formed by agave handlers from Otzumatlán and Parras, where mescal production represents a source of even higher income in the economy of all households sampled. Our result showed that their managed populations and their activity are the least vulnerable. Although they scored the higher values of risk, their diversified and complex management practices appear to be effective to mitigate that risk.

### Risk as a motive of management complexity

The regression analysis showed a positive and significant relation between these indexes, and describes and confirms our main hypothesis, that in general, the higher the risk, the greater the complexity of management responses. The handlers that are positioned near and above the regression line are in general efficient handlers that have a greater management complexity, and insofar better while more distant above of the fitted regression line, while the handlers that are below the line are in general deficient handlers, and worse as the distance below the regression line is greater. There is a particular case of some members of the second group that obtain relatively high values of risk and that are not generating enough management responses. In these communities there is a high rate of migration of people to the U.S.A., and their own testimony indicates that both wild populations and mescal production are endangered mainly because their management practices are poor. This vulnerability may be related to loss of TEK associated to migration as it has been documented in other studies [35], but this hypothesis requires further studies.

Our work confirms that, similarly as other studies have documented [3, 20, 36], the main risk factors influencing how intense are management responses are related with economic and cultural importance, particularly the pressure determined by the demand in the markets that affects directly in the resource availability, the implementation or not of harmful practices, as well as being part of a partnership. Handlers of Otzumatlán, Parras and Cañada del Agua carry on the most intense manage practices from *in situ* management forms to agrochemical dependent *ex situ* cultivation and their mescal has an increasing demand in the regional market, especially in the city of Morelia, Michoacán. In this city, during the last 10 years several specialized bars called “mezcalerías” have opened their doors to sell traditional mescal mainly from all over Michoacán and their sales and popularity are increasing day by day. We believe that the development of this promising activity may be compromised if agave handlers do not reorganize the need of practicing management strategies and include people of their communities to make decisions on the conservation of a common resource that is essential in their cultural and economic development [36]. This work analyzed the situation from only 5 of 29 municipalities of Michoacán that produces mescal, and only with the management and risk situation of one of the five agave species that are used for mescal production in this particular state. Therefore, studies in the whole context are still needed.

However, the diversity of management practices and strategies documented, may significantly contribute to the sustainability of agave use. Particularly important are the different forms of *in situ* management, which allow the conservation of biodiversity and ecosystems functions, and are in addition agrochemicals free. However, the intensive plantations through organic methods are possible and some of the agave handlers are experimenting in that direction.

It is greatly important that the academic sector supports with research the efforts that the traditional handlers are experimenting. Also important is to develop effective mechanisms of knowledge exchange and transmission of management experiences, particularly those of the most efficient handlers of this important plant resource. This type of actions may help to make shorter the route to develop sustainable extractive activities in the particular region studied, but also in other rural communities of the Mexican territory that depends on wild resources. Collaboration of research groups and the facilitation of governmental programs to enhance sustainable practices are crucial to achieve this management goal.

## Conclusions

*Agave inaequidens* represents a multiple source of benefits and potential uses not only in Michoacán but in other regions where this plant species is naturally distributed. According to the people´s perception the wild populations have decreased in quality and quantity in 30 years. The mescal demand is increasing, and therefore, the implementation of community based management strategies are needed to achieve sustainable management goals of a common resource.

Our study confirms that mostly economic pressures and availability in relation to the social demand are relevant issues that determined management responses and provides a novel approach for analyzing how meaningful these factors for encouraging management.

Maintenance of the creative dynamics of traditional ecological and transmission of the experiences and knowledge are crucial for constructing sustainable management. Promoting knowledge interchange between communities, social organizations, and regions are valuable strategies for achieving sustainable practices. Enhancing of *in situ* forest management strategies is crucial to conserve agave resources together with its biodiversity and environmental services, and the use of agrochemicals can be avoided. The interaction among mescal producers, agave handlers, governmental and non-governmental organizations and the academic sectors constructing schemes of adaptive management and fair markets are crucial for success in sustainable management of mescal and other non-timber forest products.

## Consent

Written informed consent was obtained from the people interviewed for the publication of this report and any accompanying images.
